# Structures of Merkel Cell Polyomavirus VP1 Complexes Define a Sialic Acid Binding Site Required for Infection

**DOI:** 10.1371/journal.ppat.1002738

**Published:** 2012-07-26

**Authors:** Ursula Neu, Holger Hengel, Bärbel S. Blaum, Rachel M. Schowalter, Dennis Macejak, Michel Gilbert, Warren W. Wakarchuk, Akihiro Imamura, Hiromune Ando, Makoto Kiso, Niklas Arnberg, Robert L. Garcea, Thomas Peters, Christopher B. Buck, Thilo Stehle

**Affiliations:** 1 Interfaculty Institute of Biochemistry, University of Tuebingen, Tuebingen, Germany; 2 Department of Chemistry, University of Luebeck, Luebeck, Germany; 3 Laboratory of Cellular Oncology, National Cancer Institute, Bethesda, Maryland, United States of America; 4 Department of Molecular, Cellular, and Developmental Biology, and the Biofrontiers Institute, University of Colorado at Boulder, Boulder, Colorado, United States of America; 5 National Research Council Canada, Institute for Biological Sciences, Glycobiology Program, Ottawa, Ontario, Canada; 6 Institute for Integrated Cell-Material Sciences (iCeMS), Kyoto University, Kyoto, Japan; 7 Department of Applied Bioorganic Chemistry, Gifu University, Gifu, Japan; 8 Division of Virology, Department of Clinical Microbiology, Umeå University, Umeå, Sweden; 9 Department of Pediatrics, Vanderbilt University School of Medicine, Nashville, Tennessee, United States of America; University of Michigan, United States of America

## Abstract

The recently discovered human Merkel cell polyomavirus (MCPyV or MCV) causes the aggressive Merkel cell carcinoma (MCC) in the skin of immunocompromised individuals. Conflicting reports suggest that cellular glycans containing sialic acid (Neu5Ac) may play a role in MCPyV infectious entry. To address this question, we solved X-ray structures of the MCPyV major capsid protein VP1 both alone and in complex with several sialylated oligosaccharides. A shallow binding site on the apical surface of the VP1 capsomer recognizes the disaccharide Neu5Ac-α2,3-Gal through a complex network of interactions. MCPyV engages Neu5Ac in an orientation and with contacts that differ markedly from those observed in other polyomavirus complexes with sialylated receptors. Mutations in the Neu5Ac binding site abolish MCPyV infection, highlighting the relevance of the Neu5Ac interaction for MCPyV entry. Our study thus provides a powerful platform for the development of MCPyV-specific vaccines and antivirals. Interestingly, engagement of sialic acid does not interfere with initial attachment of MCPyV to cells, consistent with a previous proposal that attachment is mediated by a class of non-sialylated carbohydrates called glycosaminoglycans. Our results therefore suggest a model in which sialylated glycans serve as secondary, post-attachment co-receptors during MCPyV infectious entry. Since cell-surface glycans typically serve as primary attachment receptors for many viruses, we identify here a new role for glycans in mediating, and perhaps even modulating, post-attachment entry processes.

## Author Summary

Viruses must interact with specific receptor molecules on their host cells in order to first attach to the cell and second gain entry into it. Therefore, a viral entry pathway is a sequence of precisely regulated binding events between viral proteins and their cellular receptors, which can be proteins or other biomolecules. In the present study, we investigated the human Merkel cell polyomavirus (MCPyV or MCV) and show that it uses complex carbohydrates containing sialic acid as receptors for entry. MCPyV was discovered in 2008, is widespread in humans and can cause aggressive skin tumors termed Merkel cell carcinomas in immunosuppressed individuals. We determined the crystal structures of the MCPyV capsid protein bound to sialylated carbohydrates, describing the contacts needed for receptor recognition in molecular detail. When we introduced targeted mutations that abolished sialic acid binding into the virus, it was unable to infect cells although it could still attach to them. Earlier studies showed that the virus uses a different group of carbohydrates called glycosaminoglycans for initial attachment to the cell surface. Thus, its entry pathway involves sequential binding to two distinct classes of carbohydrates. Our structures can be used as a starting point to develop antivirals against MCPyV.

## Introduction

The human Merkel cell polyomavirus (MCPyV or MCV) was discovered in 2008 and found to be clonally integrated into Merkel cell carcinomas (MCCs), establishing it as the first human oncovirus from the polyomavirus family [1]. MCPyV infection is common, with 50–80% of adults being seropositive [2]. It establishes persistent asymptomatic infections in the skin of healthy individuals, many of whom chronically shed virions [3]. In immunocompromised individuals, MCCs arise from the malignant transformation of mechanoreceptor Merkel cells in the skin by the transforming antigens of MCPyV [4], [5]. MCC is lethal, with an overall 5-year survival of MCC of only 50%, and its incidence has increased to 1,500 new cases per year in the USA alone [6], [7]. There are no vaccines or antivirals against MCPyV.

Polyomaviruses are non-enveloped, double-stranded DNA viruses that infect mammals and birds. There are currently nine human polyomaviruses, seven of which have been identified in the last five years [3], [8], [9], [10], [11], [12]. Similar to MCPyV, the human BK and JC Polyomaviruses (BKPyV and JCPyV) establish persistent asymptomatic infections but cause severe disease in immunosuppressed individuals [2]. Although polyomaviruses such as Simian Virus 40 (SV40) and Murine Polyomavirus (mPyV) can transform cells in culture or cause tumors in animal models, MCPyV is the first virus in the family that has been clearly implicated as a causal agent underlying a human cancer [1], [4], [5], [13].

Polyomavirus infectious entry is initiated by the major capsid protein VP1, which attaches to cellular receptors to promote internalization and transport of the viral genome into the nucleus for replication [14]. MCPyV uses sulfated carbohydrates termed glycosaminoglycans (GAGs) as attachment receptors. This contrasts with better-studied polyomaviruses, such as murine polyomavirus (mPyV), SV40, BKPyV and JCPyV, which use carbohydrates containing sialic acid for cell attachment and internalization [15], [16], [17]. Sialic acids have nevertheless been implicated in MCPyV infection as cell lines lacking sialylated glycans are resistant to transduction with an MCPyV reporter virus [18]. MCPyV VP1 has also been shown to interact *in vitro* with the ganglioside GT1b, which carries three sialic acids [19]. However, it was not understood in which way sialic acids are involved in MCPyV infection, nor whether a direct interaction with sialic acid is required for productive infection. Sialic acids cap *N*- and *O*-linked glycoproteins as well as glycolipids and are found on all eukaryotic cell surfaces. The most common sialic acid in humans is *N*-acetyl neuraminic acid (Neu5Ac) [20], [21]. Structural studies of VP1-receptor complexes from other polyomaviruses have elucidated their interactions with different sialylated oligosaccharides [17], [22], [23]. However, a putative MCPyV sialic acid binding site must differ from previously characterized ones as MCPyV lacks conserved residues that engage sialic acids in other polyomaviruses. Thus, the structural basis of MCPyV's requirement for sialylated glycans remains unknown.

In this study, we present crystal structures of MCPyV VP1 in complex with sialylated oligosaccharides. Analysis of the observed interactions in solution using NMR spectroscopy allows us to identify a linear Neu5Ac-α2,3-Gal disaccharide as the motif recognized by MCPyV. Based on the structural information, we conduct mutagenesis experiments that directly establish the functional relevance of the interaction with sialic acid for MCPyV infection. Our results therefore illuminate a crucial post-attachment interaction event of MCPyV, providing a foundation for the development of antiviral strategies.

## Results

### Overall structure of MCPyV VP1

**Figure 1:**
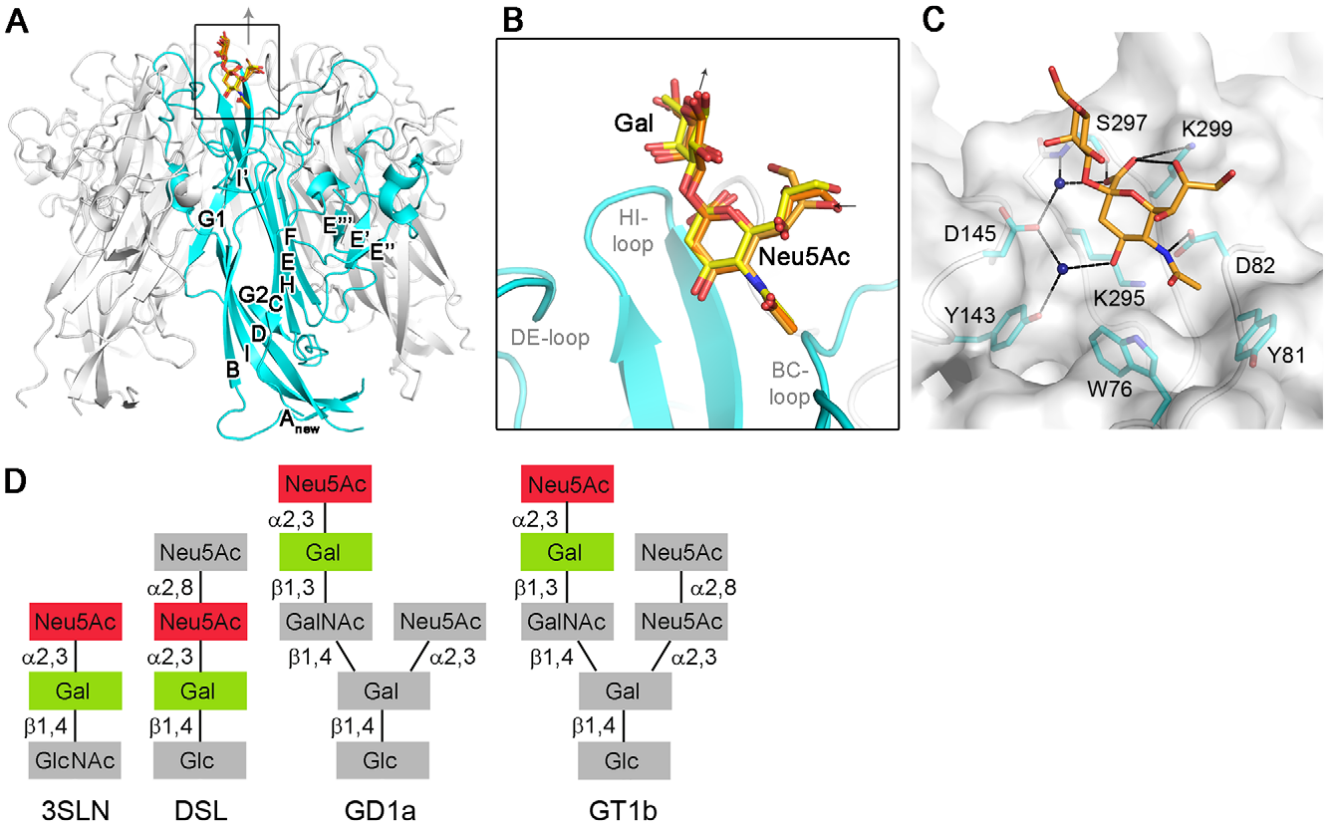
Structure of MCPyV VP1 in complex with sialylated oligosaccharides. (A, B) The protein is shown in cartoon representation, with one VP1 monomer highlighted in cyan and the other monomers depicted in gray. The oligosaccharides are drawn as stick models. Nitrogen and oxygen atoms are colored blue and red, respectively, and carbon atoms are colored orange for 3SLN, light orange for DSL and yellow for GD1a. (C) Interactions between MCPyV VP1 and the Neu5Ac-α2,3-Gal motif of DSL. The protein surface is colored grey, the protein backbone is depicted in cartoon representation and colored grey, and side chains interacting with the ligand are shown in stick representation and colored by element. Hydrogen bonds and salt bridges are shown as dashed lines, and water molecules mediating hydrogen bonds are indicated as dark blue spheres. (D) Schematic representation of the oligosaccharide structures used in this study. The binding epitope is highlighted in color.

We solved the crystal structure of unassembled MCPyV VP1 pentamers at 2.1 Å resolution (Table 1). The crystallized VP1 construct was truncated at the C-terminus to prevent VP1 assembly into capsids, and at the N-terminus to remove potentially disordered residues that inhibit crystallization. However, the construct contained the entire pentameric core of VP1. Similar truncations did not have an effect on the receptor binding properties of other polyomavirus VP1 proteins [17], [22], [23], [24]. MCPyV VP1 is a symmetric ring-shaped homopentamer with the five VP1 monomers arranged around a central five-fold axis (Fig. 1A). Each monomer is composed of two antiparallel β-sheets, which together form a β-sandwich with jelly-roll topology. With β-strands named alphabetically from the N-terminus, the two sheets consist of strands B, I, D, G, and C, H, E, F, respectively. The β-strands are linked by extensive loops that cover the top and sides of the pentamer. The apical loops, which make up the top surface of the pentamer and thus the outer surface of the virus, are the most variable parts among VP1 sequences from different polyomaviruses, creating unique interaction surfaces.

**Table 1:** Data collection and refinement statistics.

	MCPyV VP1		MCPyV VP1+DSL		MCPyV VP1+3SLN		MCPyV VP1+GD1a	
**Data collection** *								
Space group	P1		P1		P1		P1	
Cell dimensions	85.27, 85.62, 248.17		85.27, 85.77, 248.71		85.04, 85.70, 248.73		84.66, 130.15, 165.53	
*a*, *b*, *c* (Å)								
α, β, γ (°)	92.98, 100.50, 108.05		92.97, 100.48, 108.05		93.02, 100.41, 108.07		98.04, 101.02, 105.80	
Resolution (Å)+	45 – 2.1	(2.15 – 2.10)	50 – 1.85	(1.90 – 1.85)	50 – 2.0	(2.05 – 2.00)	50 – 2.4	(2.46 – 2.40)
*R*_merge_ (%)	11.3	(56.9)	9.6	(61.2)	8.5	(47.1)	10.1	(66.6)
*I*/σ*I*	9.0	(2.9)	9.5	(1.7)	8.2	(1.6)	8.7	(2.1)
Completeness (%)	92.3	(64.7)	94.7	(90.0)	89.0	(77.0)	97.3	(96.8)
Redundancy	2.8	(2.7)	3.3	(2.9)	2.1	(1.9)	3.6	(3.7)
**Refinement**								
Resolution (Å)	45 – 2.1	(2.15 – 2.10)	50 – 1.85	(1.90 – 1.85)	50 – 2.0	(2.05 – 2.00)	50 – 2.4	(2.46 – 2.40)
No. reflections	351,188	(18,198)	529,047	(37,360)	395,073	(25,120)	248,679	(18,243)
*R*_work_/*R*_free_	18.1/22.6	(25.4/28.4)	16.5/19.5	(25.9/29.9)	19.5/23.5	(29.6/33.0)	19.4/24.7	(27.4/33.0)
No. of atoms								
Protein	42,888		43,792		43.119		42,581	
Carbohydrate	—		224		120		213	
Solvent	3,540		4,936		3,864		1,657	
*B*-factors								
Protein	23.9		19.2		30.1		38.5	
Carbohydrate	—		29.3		41.0		50.6	
Solvent	24.9		27.4		26.7		33.1	
R.m.s. deviations								
Bond lengths (Å)	0.008		0.008		0.008		0.008	
Bond angles (°)	1.1		1.1		1.1		1.1	

*: One crystal was used for each dataset.

**+:** Values in parentheses are for the highest-resolution shell.

### Structure of MCPyV-VP1 oligosaccharide complexes

We next determined high-resolution structures of MCPyV VP1 in complex with three different sialylated oligosaccharides derived from the *in vitro* binding partner GT1b (Fig. 1). 3′-Sialyllactosamine (3SLN) is a linear compound containing a single α2,3-linked Neu5Ac residue (Fig. 1D). Disialyllactose (DSL) is also linear, carrying a second, α2,8-linked Neu5Ac attached to the one present in 3SLN. Both 3SLN and DSL are similar to the carbohydrate portions of gangliosides (GM3 and GD3, respectively), but they also can be found capping the carbohydrate parts of glycoproteins. GD1a, the oligosaccharide portion of the GD1a ganglioside, is a branched compound containing two α2,3-linked Neu5Ac residues, one branching and one linear (Fig. 1D). It is a carbohydrate sequence uniquely found on gangliosides. Tight crystal packing prevented us from obtaining a complex with the larger GT1b oligosaccharide, which had been shown to interact with MCPyV VP1 *in vitro* [19]. For simplicity, the ganglioside nomenclature will be used with respect to the GD1a and GT1b oligosaccharides from here on.

In each complex, electron density was only observed for the Neu5Ac-α2,3-Gal motif that is common to all three investigated oligosaccharides and is also present in GT1b (Fig. S1A–C). This disaccharide motif binds to a shallow binding site on the outer surface of VP1, which is formed entirely by residues of the BC-, DE- and HI-loops of one VP1 monomer (Fig. 1B,C). In all complexes, some binding sites are blocked by crystal contacts and thus not occupied, while others have bound the carbohydrate ligand, all in an identical manner (Fig. 1B). There were some binding sites in each complex that were only weakly occupied, and into which the ligands were not modeled. In all instances where ligand was bound, the α2,3-glycosidic linkage between Neu5Ac and Gal adopts the same conformation (torsion angles of −54° and −6°) that is preferred in solution and that is also found in complexes of mPyV VP1, hemagglutinins of influenza A viruses and wheat-germ agglutinin with linear α2,3-sialylated oligosaccharides [23], [25], [26]. The structure of MCPyV VP1 bound to the oligosaccharides is virtually identical to the unbound state, indicating that the carbohydrates dock into a preformed binding pocket.

### MCPyV VP1 interactions with sialylated carbohydrates

Neu5Ac forms the major contact point with MCPyV as it contributes most interactions and is best defined by electron density (Fig. S1A–C). Most of its protruding functional groups are engaged by the protein. Its carboxyl group forms a salt bridge and hydrogen bonds with K299 and S297 in the HI-loop and water-mediated hydrogen bonds with D145 and S297 (Fig. 1C). The Neu5Ac *N*-acetyl group faces away from the fivefold axis, interacting with residues in the BC-loop. It makes hydrophobic interactions with the side chains of W76 and Y81 and hydrogen bonds with D82, which forms a salt bridge with K295. Furthermore, the O4 hydroxyl group of Neu5Ac interacts with Y143 and D145 in the DE-loop via a water molecule. The glycerol chain of Neu5Ac faces away from the VP1 surface, and more than one conformation was observed for this chain in our complexes. Contacts with Neu5Ac are mostly mediated by side chains and all residues directly interacting with Neu5Ac are strictly conserved among MCPyV isolates and among newly identified MCPyV-like viruses of great apes [27] (Fig. S2). The Gal residue does not contact the protein directly and exhibits elevated temperature factors, but can be docked unambiguously into the electron density. It is likely stabilized by the conformational preferences of the glycosidic bond.

### Carbohydrate epitope mapping

**Figure 2:**
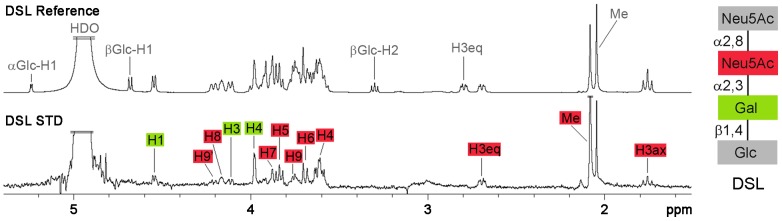
MCPyV VP1 epitope mapping of DSL. ^1^H-NMR-spectra showing the binding epitope of DSL binding to MCPyV VP1 in solution. Top: DSL reference spectrum, bottom: STD spectrum in the presence of MCPyV VP1. Assignments are color-coded according to the DSL cartoon. Signals in the STD spectrum belong to the Neu5Ac-α2,3-Gal motif in DSL only. One methyl group and residual HDO peaks are truncated. Spectra were recorded at 283 K.

Only the Neu5Ac-α2,3-Gal motif is clearly defined in our electron density maps, suggesting that this disaccharide unit serves as the main MCPyV binding target. In the DSL complex, only the internal Neu5Ac-α2,3-Gal sequence is contacted by the protein, while the terminal α2,8-linked Neu5Ac does not have clear electron density and is therefore flexible (Fig. 1D, Fig. S1A). To probe the interaction of MCPyV VP1 with DSL in solution, we analyzed the complex using saturation transfer difference (STD) NMR spectroscopy. In this technique, saturation from a macromolecule is transferred to a small-molecule ligand, reaching only those parts of the ligand within roughly 5 Å from the protein. By inspecting the resulting STD spectrum, the macromolecule-bound parts of the ligand can be mapped. With the exception of the axial Neu5Ac proton H3 (H3_ax_), all signals from the two Neu5Ac rings resonate at different frequencies and can therefore be clearly distinguished (Fig. S3). Saturation transfer from the protein was observed only to hydrogen atoms of the internal Neu5Ac and the connecting Gal, confirming the interactions seen in all crystal structures (Fig. 2). For the terminal Neu5Ac, the equatorial H3 (H3_eq_) proton as well as H4, H5 and H6 protons receive no saturation, while all the equivalent protons for the internal Neu5Ac are observed in the STD spectrum (Fig. S3). The Neu5Ac H3_ax_ signal in the STD spectrum most likely arises from contacts with the internal Neu5Ac only. Likewise, for the Neu5Ac methyl groups, significantly more transfer is observed for the internal Neu5Ac. The relatively small saturation of the terminal Neu5Ac methyl signal is also observed in the absence of protein and due to relaxation artifacts (Fig. S3). Some saturation transfer was also observed to the glycerol chain of the internal Neu5Ac ring as well as to some Gal protons (Fig. 2). These portions of DSL are not tightly tethered to the protein in the crystal structure, but they are within the 5 Å saturation transfer limit from the VP1 surface.

We next asked whether MCPyV had a preference for one of the two Neu5Ac-α2,3-Gal motifs present in GD1a, the linear group on the “left arm” of GD1a or the branching group forming the “right arm” of the oligosaccharide (Fig. 1D). Indeed, the electron density map of the GD1a complex contains weak electron density features close to the Gal residue, which are compatible with the linear Neu5Ac-α2,3-Gal epitope, but not with the branching one (Fig. S1D). Moreover, addition of a branch to the MCPyV-bound Gal residue would result in steric clashes with the protein. Thus, we can unambiguously assign the MCPyV-binding epitope to the linear Neu5Ac-α2,3-Gal moiety on the “left arm” of GD1a.

We then analyzed the MCPyV VP1 interaction with GT1b by STD NMR, and could confirm the interaction in solution (Fig. S4). Saturation transfer was observed for the methyl group of α2,3-linked Neu5Ac as well as for at least one Gal H1 proton, indicating that the Neu5Ac-α2,3-Gal epitope is likely also recognized on GT1b. However, heavy signal overlap in the heptasaccharide rendered further assignment difficult. Like GD1a, the GT1b oligosaccharide contains two Neu5Ac-α2,3-Gal motifs, one linear and one branching. Given the specificity of MCPyV for the linear epitope on GD1a, it is highly likely that MCPyV also binds the linear Neu5Ac-α2,3-Gal motif on GT1b.

In summary, our epitope mapping establishes the linear Neu5Ac-α2,3-Gal disaccharide as the motif recognized by MCPyV VP1, which is preferred over both a terminal α2,8-linked Neu5Ac residue and a branching Neu5Ac-α2,3-Gal motif.

### Sialic acid binding is crucial for a post-attachment step of MCPyV infection

**Figure 3:**
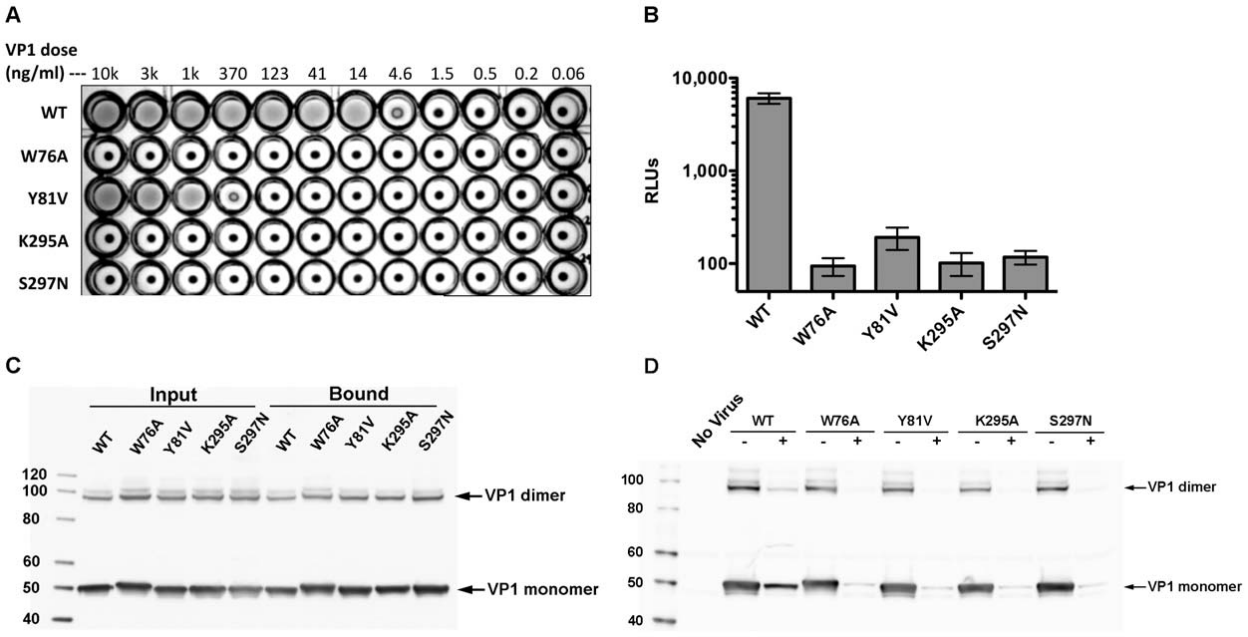
Sialic acid binding site of MCPyV VP1 is crucial for infectivity. (A) Sialic acid binding site mutantions impair hemagglutination. Hemagglutination assays were performed by mixing a suspension of sheep red blood cells with varying doses of purified wild-type (WT) or VP1 mutant MCPyV capsids. (B) Pseudovirus transduction of sialic acid binding site mutants. Human A549 cells were inoculated with WT or mutant VP1 pseudovirions carrying an encapsidated GLuc reporter plasmid. Three days after inoculation, the culture supernatants were tested for GLuc activity (measured in relative light units, RLUs). (C) Cell attachment of sialic acid binding site mutants. A549 cell suspensions were mixed with 150 ng of WT or mutant VP1 capsids for 2 hours at 4°C. The cells were then washed and subjected to Western blotting using a polyclonal serum specific for MCPyV VP1. In the lanes labeled “Input,” 50 ng of VP1 was loaded into each lane. In the lanes labeled “Bound,” 2/3^rds^ of the washed cell pellet was loaded into each lane. (D) Cell attachment of sialic acid binding site mutants depends on GAGs. A549 cell suspensions (5×10^4^/well) were treated with a mixture of heparinase I (875 mU), heparinase III (87.5 mU), and chondroitinase ABC (35 mU) or mock treated in 50 µl of digestion buffer (20 mM HEPES, pH 7.5, 150 mM NaCl, 4 mM CaCl_2_ and 0.1% BSA) for one hour at 37°C. Then, 200 ng of WT or mutant VP1 capsids diluted in 200 µl of Opti-MEM were added and the mixture was incubated for 2 hours at 4°C. The cells were then washed and subjected to Western blotting using a polyclonal serum specific for MCPyV VP1.

To probe the importance of sialic acid for MCPyV infection, we introduced mutations in the sialic acid binding site of MCPyV VP1 that either remove important interactions (W76A, Y81V, K295A) or create steric hindrance (S297N). Western blots of mutant VP1 proteins expressed in mammalian cells revealed VP1 laddering, indicating the presence of disulfide crosslinks characteristic of assembled capsids (Fig. 3D). Nuclease-digested purified stocks of wild-type and mutant capsids contained comparable amounts of encapsidated DNA, ranging from 0.12–0.14 ng of DNA per ng of VP1. Thus, the mutations are unlikely to have caused major structural changes. Recombinant MCPyV VP1 has previously been shown to bind and hemagglutinate sheep RBCs by interacting with sialylated glycans [19]. The mutant capsids showed impaired hemagglutination ability, indicating that each mutated residue is functionally involved in forming the sialic acid binding site (Fig. 3A). We then asked whether the mutant capsids are infectious. Pseudovirions generated using each of the mutant VP1s were deficient in infectious delivery of an encapsidated *Gaussia* luciferase reporter plasmid to cultured human A549 cells (Fig. 3B), demonstrating a requirement for direct interactions between the MCPyV virion and sialylated glycans during the infectious entry process. A further set of experiments examining the binding of capsids to cells revealed that each of the VP1 mutant capsids bound to A549 cells at least as efficiently as wild type VP1 (Fig. 3C). This observation is consistent with a prior report indicating that initial MCPyV-cell interactions are mediated primarily by non-sialylated GAGs [18]. The binding of each mutant was antagonized by pre-treatment of the cells with GAG-degrading enzymes (Fig. 3D), confirming that attachment is mediated by GAGs, even for the mutant capsids. Pre-treatment of cells with GAG-degrading enzymes has previously been shown to decrease wild-type MCPyV infection due to a failure of the virus to stably attach to cells [18]. Interestingly, wild-type MCPyV showed weak residual binding to cells treated with GAG-degrading enzymes, while the mutants did not (Fig. 3D). Thus, this low level of residual binding might arise from binding to sialylated oligosaccharides on host cells. As the sialic acid binding site mutants were capable of attaching to GAGs, our data demonstrate that the MCPyV GAG-binding motif is distinct from the sialic acid binding site.

### Comparison with sialic acid binding sites of other polyomaviruses

**Figure 4:**
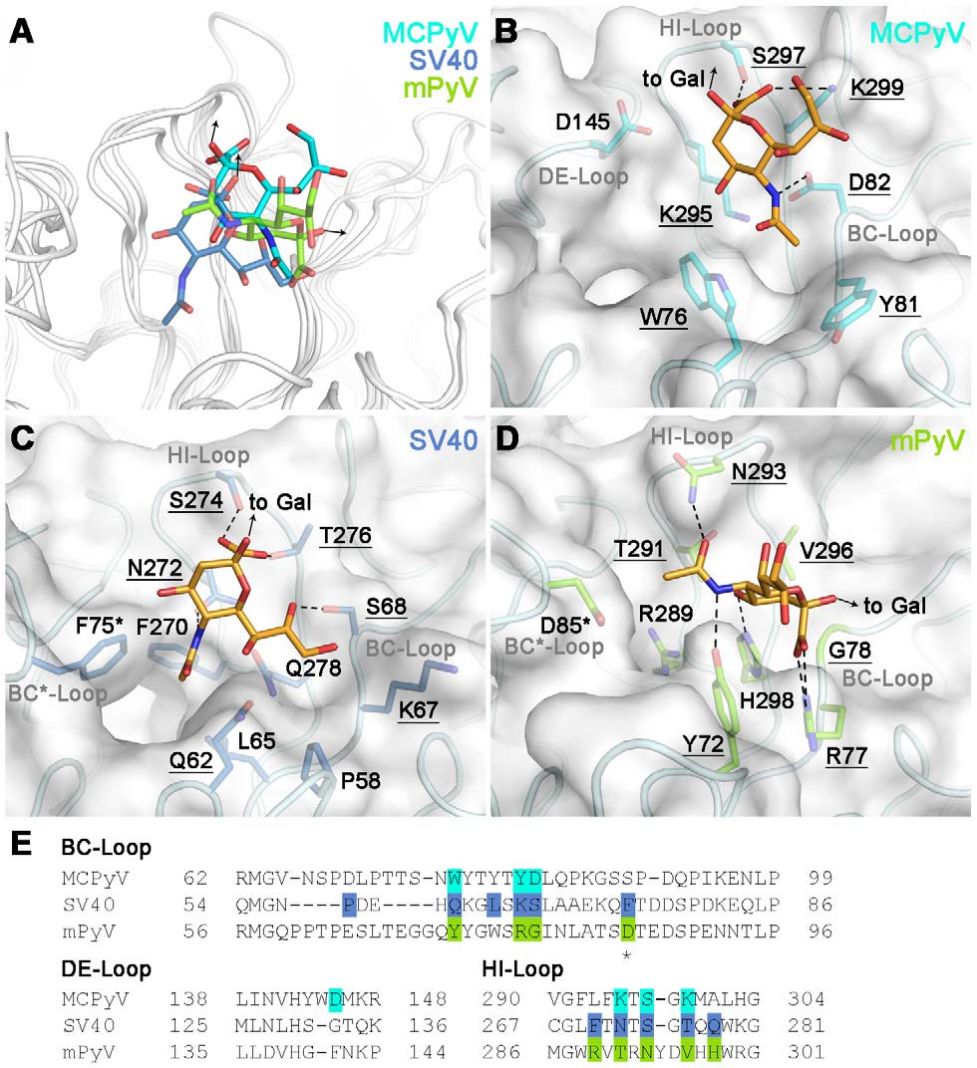
Plasticity in sialic acid recognition by polyomaviruses. (A) Overlay of ligand binding sites of MCPyV VP1 (pdb 4FMH), SV40 VP1 (pdb 3BWR) and mPyV VP1 (pdb 1VPS). The proteins are shown in cartoon representation and colored grey, while the sialic acid residues are depicted in stick representation and colored according to complex, with the MCPyV-DSL complex colored cyan, the SV40-GM1 complex colored blue and the mPyV complex colored green. The SV40 and mPyV ligands are semi-transparent. (B–D) Specific interactions of MCPyV VP1 (B), SV40 VP1 (C) and mPyV VP1 (D) with sialic acid. The protein surface is shown in grey. The protein backbones are depicted in cartoon representation, residues in contact with sialic acid are shown in stick representation and colored according to complex as in (A). Residues in equivalent positions are underlined and those contributed from a second VP1 monomer are indicated with an asterisk. Sialic acid is shown in stick representation and colored orange. Hydrogen bonds and salt bridges are shown as dashed lines. (E) Structure-based sequence alignment of the receptor-binding surface loops of MCPyV, SV40 and mPyV VP1. Residues interacting with sialic acid are highlighted as in (B–D).

Sialic acid-containing oligosaccharides serve as receptors for related polyomaviruses such as mPyV, SV40 and JCPyV. Although all of these proteins recognize different sialylated ligands, sialic acid is the main point of attachment in each case, with auxiliary interactions determining the individual binding specificities [17], [22], [23]. Interestingly, there are three entirely different Neu5Ac binding modes among the four viruses, exemplified by MCPyV, mPyV and SV40 (Fig. 4). Such a high degree of variability is unusual. Related viruses usually possess virtually identical binding sites for sialic acid, as demonstrated by comparisons of JCPyV and SV40 [17], [22], [23], or of different Influenza A viruses [28]. Specificity is in these cases achieved by augmenting contacts that surround the central, conserved sialic acid binding site and that modulate binding to different sialylated carbohydrates [17], [22], [23]. Interestingly, all but one of the MCPyV Neu5Ac binding residues are identical in similar viruses of great apes (Fig. S2), indicating that these viruses share a common sialic acid binding site.

The Neu5Ac binding sites of MCPyV, SV40 and mPyV do not lie in different regions of VP1, but rather use equivalent positions in sequence and in structure (Fig. 4B–E). However, each binding site employs a unique set of residues in these positions that in each case engage Neu5Ac in a different orientation (Table 2). In the MCPyV complex, for instance, the *N*-acetyl group of Neu5Ac points away from the fivefold axis (Fig. 4B), while it is oriented towards the clockwise neighboring monomer and inserts into a protein cavity in the SV40 complex (Fig. 4C), and faces directly towards the clockwise neighboring monomer in the mPyV complex (Fig. 4D). The *N*-acetyl group is contacted by a hydrogen bond to D82 and hydrophobic interactions with W76 and Y81 in the MCPyV complex, by a hydrogen bond to N272 and hydrophobic interactions with F270 and Q62 as well as F75* from the neighboring monomer in the SV40 complex, and by a hydrogen bond to Y72 in the mPyV complex (Table 2). Likewise, the carboxylate group of Neu5Ac is recognized by a hydrogen bond to S297 and a salt bridge to K299 in the MCPyV complex, by two hydrogen bonds to S274 and T276 in the SV40 complex and by a salt bridge to R78 in the mPyV complex (Table 2). Nevertheless, the Cα positions of the residues involved in Neu5Ac recognition are very similar in the three viruses, implying that the backbone structure in this region of VP1 is particularly suitable for evolving carbohydrate binding sites. Thus, while conserved amino acids imply that two proteins share a similar binding site, non-conserved amino acids do not exclude the possibility that two proteins engage similar carbohydrates in equivalent locations. The level of plasticity observed in the polyomavirus family could well be present in other families of carbohydrate-binding proteins where structural information is still lacking and does not yet allow for comparisons of binding modes.

**Table 2:** Variable interactions with sialic acid among polyomavirus VP1 proteins.

MCPyV		SV40		mPyV	
Residue	Interaction	Residue	Interaction	Residue	Interaction
		P58	Van der Waals interaction with C9 of glycerol chain		
W76	Van der Waals interaction with *N*-acetyl group	Q62	Van der Waals interaction with glycerol chain	Y72	Hydrogen bond to amide nitrogen of *N*-acetyl group
		L65	Van der Waals interaction with *N*-acetyl group		
Y81	Hydrophobic interaction with methyl moiety of *N*-acetyl group	K67	Van der Waals interaction with glycerol chain	R77	Salt bridge to carboxylate group
D82	Hydrogen bond to amide nitrogen of *N*-acetyl group	S68	Hydrogen bond to O8 of glycerol chain	G78	Backbone hydrogen bond to carboxylate group; accommodates adjacent monosaccharide
		D75*	Hydrophobic interaction with methyl moiety of *N*-acetyl group	D85*	Van der Waals interaction with methyl moiety of *N*-acetyl group
D145	Water-mediated hydrogen bond to carboxylate group				
		F270	Hydrophobic interaction with methyl moiety of *N-*acetyl group	R289	Water-mediated hydrogen bond to O4 hydroxyl group on ring
K295	Van der Waals interaction with *N-*acetyl group; orientation of D82	N272	Hydrogen bond to amide nitrogen of *N-*acetyl group	T291	Van der Waals interaction with *N*-acetyl group
S297	Hydrogen bond to carboxylate group	S274	Hydrogen bond to carboxylate group	N293	Hydrogen bond to amide carbon of *N*-acetyl group
K299	Salt bridge to carboxylate group	T276	Hydrogen bond to carboxylate group	V296	Van der Waals interaction with ring
		Q278	Van der Waals interaction with glycerol chain; orientation of N272	H298	Hydrogen bond to O4 hydroxyl group on ring

## Discussion

We demonstrate here that the MCPyV major capsid protein VP1 directly interacts with carbohydrates bearing a linear Neu5Ac-α2,3-Gal motif. Our high-resolution structures of complexes reveal the molecular interactions governing recognition of this motif. The observed interaction has functional relevance as VP1 point mutants that lack sialic acid binding capability are unable to mediate infectious delivery of an encapsidated reporter plasmid to host cells. As these mutations did not affect GAG-dependent attachment, the sialic acid binding site described here must be functionally distinct from the as yet unidentified GAG-binding site on the virion surface.

The idea that MCPyV infectious entry requires a direct interaction with a sialylated cellular glycan for an entry step that takes place after stable GAG-dependent attachment to the cell helps reconcile a prior report by Erickson and colleagues, who postulated that MCPyV infectious entry requires a direct binding interaction with sialylated glycans [19], with a subsequent report by Schowalter and colleagues indicating that sialylated glycans are not required for MCPyV attachment to cultured cell lines [18]. A requirement for direct interactions between MCPyV VP1 and a sialylated glycan for a post-attachment infectious entry step also explains the past observation that Lec2 cells, which are deficient in biosynthesis of sialylated glycans, readily bind MCPyV but nevertheless do not support MCPyV infectious entry unless sialylated glycan synthesis is restored [18].

The treatment of cells with neuraminidases allows conclusions about viral entry in most cases. For example, neuraminidase treatment of cells dramatically reduces the infectivity of BKPyV, which uses gangliosides such as GT1b as sole receptors for both attachment and entry [15]. The infectivity of JCPyV, which uses the sialylated glycan LSTc as a primary receptor, is likewise sensitive to neuraminidase [17], [29]. However, it can be difficult to determine, by neuraminidase treatment alone, whether viruses depend on sialylated glycans for entry. In fact, MCPyV infectivity does not appear to be affected by transient neuraminidase treatment [18]. An explanation for this finding is that MCPyV can use GAG-dependent binding to stably persist on the cell surface until neuraminidase activity wanes and newly synthesized sialylated glycans begin to reappear on the cell surface. In light of this explanation, it seems possible that sialic acid is used as a secondary receptor by other viral species, although such usage of sialylated glycans as post-attachment co-receptors is essentially unheard of among viruses investigated to date [30]. For some of these viruses, the need for a sialylated glycan co-receptor may have not yet been uncovered because engagement of the primary receptor influences the outcome of transient neuraminidase experiments, thus masking the involvement of sialic acids.

Our data establish a linear Neu5Ac-α2,3-Gal disaccharide as a specific MCPyV binding motif. We have confirmed the interaction for the 3SLN, DSL, GD1a and GT1b oligosaccharides, which all contain this motif. Our data agree well with the observation that the ganglioside GT1b interacts with MCPyV VP1 pentamers in sucrose flotation assays [19]. However, it differs from the earlier observation that GD1a did not bind MCPyV VP1 [19]. We can exclude that GD1a binding in our experiments was mediated by crystal contacts as no symmetry-related VP1 molecules were bridged by GD1a. It is possible that the use of GST-tagged VP1 pentamers, which can form large aggregates by dimerization of GST-tags, interfered with GD1a binding in the earlier study.

Linear Neu5Ac-α2,3-Gal disaccharides are present on many different classes of carbohydrates, such as gangliosides and other glycolipids, *N*- and *O*-linked glycoproteins [20]. As we do not see any contacts of MCPyV with carbohydrate residues outside the binding epitope in our structures, it is likely that MCPyV can bind to most oligosaccharides bearing a linear Neu5Ac-α2,3-Gal disaccharide. The MCPyV binding epitope is thus smaller and present on more oligosaccharides than other sialylated polyomavirus ligands, such as the SV40 receptor GM1 and the JCPyV receptor LSTc, which are both recognized with higher specificity [17], [22]. In contrast, MCPyV likely binds sialylated oligosaccharides in a more promiscuous manner, similar to the well-characterized mPyV, whose receptor interactions have interesting parallels to MCPyV. First, the mPyV binding epitope is also the linear Neu5Ac-α2,3-Gal disaccharide [23], [31], and second, mPyV can bind to several different oligosaccharides bearing that motif. Notably, only few of them, the gangliosides GD1a and GT1b, are known to mediate mPyV infection [16], [32], [33], while other carbohydrates present on glycoproteins are hypothesized to be ‘pseudoreceptors’ for mPyV that bind the virus but do not mediate infection [33], [34]. Gangliosides were therefore early MCPyV receptor candidates, especially because the ganglioside GT1b was known to interact with MCPyV in vitro [19]. However, GT1b supplementation of ganglioside- or sialyl glycan-deficient cells did not rescue MCPyV infection, while it did rescue the infectivity of BKPyV [18] (RMS and CBB, unpublished data). Thus, GT1b or other gangliosides are unlikely to serve as functional secondary receptors for MCPyV. We think it likely that MCPyV is able to bind many sialylated oligosaccharides on host cells and that, similar to mPyV, both functional and pseudoreceptors regulate entry processes. However, further studies will be needed to elucidate the roles of the various glycans bearing linear Neu5Ac-α2,3-Gal disaccharides in MCPyV infection, and to define which are the functional secondary receptors and which act as pseudoreceptors. This model does not exclude the possibility that MCPyV might engage an as yet unidentified longer oligosaccharide bearing a binding epitope with additional contacts and therefore serving as a higher affinity secondary receptor. However, the examples of mPyV and of human Influenza A viruses show that despite promiscuous binding properties, a higher affinity receptor may not be required [28], [33].

Taken together with previous studies, our findings strongly support a novel uptake pathway in which infectious entry of MCPyV requires both initial attachment to GAGs and subsequent interaction with a sialylated oligosaccharide. Our structural and functional data allow conclusions about several of the steps involved. Entry is initiated by attachment of MCPyV to GAGs on the cell surface, which is likely mediated by the major capsid protein VP1 as there is no indication in polyomavirus structures of exposed minor capsid proteins [31], [35]. Since our mutant pseudovirions deficient in sialic acid binding still were capable of GAG-mediated attachment, the two binding sites are separated entities on the VP1 surface, and sialic acids and GAGs do not use a dual function binding site. However, our structural data do not allow prediction of the GAG binding site as there is no conservation of GAG binding among polyomaviruses. Attachment to GAGs does not seem to be a prerequisite for sialic acid binding, as the protein clearly bound sialylated ligands in the absence of GAGs (Figs. 1, 2). Instead, we think it likely that the initial attachment to GAGs helps to concentrate viral particles at or close to the cell surface, perhaps compensating for the relatively low affinity of the MCPyV VP1-sialic acid interaction. The *K*_D_ value of this interaction must be in the mM range because the STD NMR experiment only covers µM-mM interactions [36], and oligosaccharide concentrations in the mM range were necessary to obtain complex crystals.

While the MCPyV-GAG interaction is well characterized on the functional level, but not structurally, it is the other way round for the interaction with sialic acid. Two key questions remain. First, which of the many oligosaccharide moieties bearing the epitope described here is the functional secondary receptor for MCPyV? And second, what is its role during MCPyV entry? One enticing possibility is that the sialic acid-dependent step in MCPyV entry mediates intracellular trafficking. Different polyomaviruses use differing machineries for initial uptake, such as cholesterol-mediated endocytosis for SV40, BKPyV and mPyV, or clathrin-dependent endocytosis in the case of JCPyV [37], [38]. However, trafficking after uptake appears to converge en route to the ER, and to depend on sialylated glycans at least in some cases [16], [33], [39], [40]. MCPyV might use similar trafficking routes after GAG-dependent attachment. However, more functional studies will be necessary to define its role, and the point mutants in the sialic acid binding site we describe here might be interesting tools in this investigation.

Importantly, the MCPyV uptake pathway differs from those of other polyomaviruses investigated to date, none of which require GAGs for infection [15], [16], [29], and from papillomavirus entry pathways, which depend on GAGs, but do not need sialic acid [14]. It also differs from the receptor requirements of some adeno-associated viruses that are able to bind both GAGs and sialic acids, but can use either one as receptors [41], and do not require ordered, direct interactions with both. The present findings may also inform the study of other viruses because it is quite likely that MCPyV is not the only virus to rely on both GAGs and sialic acid to infect cells.

In conclusion, we have established a specific interaction of MCPyV VP1 with a linear sialylated disaccharide, and demonstrate the functional relevance of this interaction for MCPyV infection. The observed interactions provide a useful platform for the development of MCPyV-specific vaccines and antivirals. The novel uptake mechanism of MCPyV, requiring GAGs and sialic acid sequentially, furthermore enhances understanding of cell entry by carbohydrate-engaging viruses. Comparison with other polyomavirus-receptor structures demonstrates the high level of adaptation that sialic acid binding sites can undergo, informing both viral and non-viral protein-carbohydrate recognition processes.

## Materials and Methods

### Carbohydrate production

Two different GT1b oligosaccharide compounds with different linkers on the terminal glucose were used in this study for crystallization and NMR experiments. The first compound (1) was synthesized as described [42] and carries a CH_2_-CH_2_-CH_2_-N_3_ linker at the anomeric carbon of the Glc ring. The second compound (2) was synthesized from the lactose derivative 2-(trimethylsilyl)ethyl 2,6-di-*O*-benzyl-β-D-galactopyranosyl-(1→4)-2,3,6-tri-*O*-benzyl-β-D-glucopyranoside using the reaction scheme described by Ishida *et al.* [43], [44]. It carries a CH_2_-CH_2_-Si(CH_3_)_3_ linker on the anomeric carbon of the Glc ring. In both compounds, the linkers inhibit mutarotation between α- and β-Glc. GD1a oligosaccharide was produced by ozonolysis of GD1a ganglioside as described [45], [46].

### Protein expression and purification

DNA coding for amino acids 38–320 of w162 MCPyV VP1 (GenBank # FJ392560) was amplified by PCR and cloned into the pET15b expression vector (Novagen) in frame with an N-terminal hexahistidine tag (His-tag) and a thrombin cleavage site. The protein was overexpressed in *E. coli* BL21(DE3) and purified by nickel affinity chromatography and gel filtration on Superdex-200 (GE Healthcare). The tag was cleaved with thrombin prior to gel filtration, leaving the non-native amino acids GSHMLE at the N-terminus.

### Crystallization and complex formation

MCPyV VP1 was in a buffer comprised of 20 mM HEPES pH 7.5, 150 mM NaCl and 20 mM DTT after gel filtration. The protein was concentrated to 3.5 mg/mL and crystallized at 20°C by hanging drop vapor diffusion against a reservoir solution containing 100 mM sodium cacodylate pH 6.5, 6% (w/v) PEG 3,350 and 300 mM magnesium chloride. A seeding stock was included in the crystallization drops. Crystals were harvested into reservoir solution, cryoprotected by soaking them in reservoir solution supplemented with 25% (v/v) glycerol for 10 s, and flash-frozen in liquid nitrogen. For oligosaccharide complex formation, crystals were soaked in reservoir solution supplemented with 20 mM DSL (Sigma), 25 mM GD1a oligosaccharide or 20 mM 3SLN (Sigma) for 10–40 min. The same concentration of oligosaccharide was also included in the cryoprotection solution. Similar approaches to cocrystallization were used for unsuccessful attempts to obtain complexes with GT1b oligosaccharides.

### X-ray structure determination

All diffraction data were collected at −180°C and at a wavelength of 1 Å. Datasets for native and complexed MCPyV VP1 were recorded at beamlines 14.1 at BESSY (Berlin, D) and X06DA at SLS (Villigen, CH), respectively (Table 1). X-ray data were processed with xds [47], and the structure was solved by molecular replacement with Molrep [48] using a search model generated from the structure of mPyV VP1 (pdb 1VPN) [23]. Each structure contained four VP1 pentamers in the asymmetric unit. The structures were completed by iterating rounds of model building in Coot, and with simulated annealing, restrained coordinate and B-factor as well as TLS refinement in Refmac5 and Phenix [49], [50], [51]. Initial TLS parameters were generated using the TLSMD server [52]. The 20-fold non-crystallographic symmetry linking the VP1 monomers in the asymmetric unit was used as a restraint throughout refinement for protein regions outside crystal contacts. Oligosaccharide ligands were located in weighted mF_o_-DF_c_ difference electron density maps, and refined using restraints from the Refmac library and user-defined ones for the glycosidic linkages of sialic acid. The final models agree well with the experimental data and have good geometry (Table 1). In all models, more than 95.5% of amino acids are in the favoured region of the Ramachandran plot, with <0.1% in the disallowed regions. Coordinates and structure factor amplitudes for the native, DSL complex, 3SLN complex and GD1a complex MCPyV VP1 structures were deposited in the PDB under accession codes 4FMG, 4FMH, 4FMI, and 4FMJ, respectively.

### STD NMR measurements

NMR spectra were recorded at 283 K using 3 mm tubes on a Bruker DRX 500 MHz spectrometer fitted with a 5 mm cryogenic probe (DSL interaction) or on a Bruker AVIII-600 spectrometer equipped with a room temperature probehead (GT1b interaction), and processed with TOPSPIN 2.0 (Bruker). A sample containing 42 µM MCPyV VP1, 1 mM DSL (Sigma), 20 mM deutero-Tris pH 7.5, 20 mM deutero-β-mercaptoethanol, 150 mM NaCl was used for the STD NMR analysis of the DSL-VP1 interaction. For the GT1b-VP1 interaction, a sample containing 26 µM MCPyV VP1 and 2 mM GT1b was prepared using the same buffer as for the DSL-containing sample. Samples containing 1 mM DSL or 2 mM GT1b but no protein were prepared and were used for spectral assignment and to confirm that the chosen on-resonance frequencies did not directly excite the ligands. Samples were prepared in D_2_O and no additional water suppression was used in order not to affect the anomeric proton signals. The off- and on-resonance frequencies were set to 80 ppm and 7 ppm, respectively. The total relaxation delay was 4 s. A cascade of 40 Gaussian-shaped pulses with 50 ms duration each, corresponding to a strength of 65 Hz, and a saturation time of 2 s was used for selective excitation. A 10 ms continuous-wave spin lock filter with a strength of 3.7 kHz was employed in order to suppress residual protein signals. 32 k points were collected and zero filling to 64 k data points was employed. Spectra were multiplied with an exponential line broadening factor of 2 Hz prior to Fourier transformation. Spectra were referenced using HDO as an internal standard [53]. Pure oligosaccharide samples containing 1 mM DSL or 2 mM GT1b served as samples for spectral assignment. Series of 1D ^1^H-TOCSY and COSY spectra as well as ^1^H, ^13^C-HSQC spectra were acquired for assignment of the oligosaccharide proton resonances. Literature values on related oligosaccharides served as assignment controls [54], [55], [56].

### VP1 mutagenesis and functional testing

A previously-reported system for production of MCPyV-based reporter vectors (pseudoviruses) [57] was used for functional analysis of mutant VP1 proteins. Briefly, expression constructs carrying codon-modified ORFs encoding MCPyV VP1 and VP2 proteins were co-transfected into 293TT cells along with a reporter plasmid encoding *Gaussia* luciferase. The resulting MCPyV pseudovirions were released from the transfected cells by detergent lysis in the presence of a DNase/RNase cocktail. The pseudovirions were allowed to mature overnight, then purified by ultracentrifugation through Optiprep gradients. Gradient fractions were screened for the presence of encapsidated DNA using Picogreen reagent (Invitrogen). VP1 content of the purified reporter vector stocks was standardized based on SYPRO Ruby (Invitrogen) stained SDS-PAGE gel analysis. HA assays, infectivity assays and cell binding assays as well as heparinase/chondroitinase treatment were carried out as previously reported [18].

## Supporting Information

Figure S1 **Electron density for carbohydrate ligands.** Simulated annealed omit difference electron density maps for DSL (A), 3SLN (B) and GD1a (C,D). In A–C, the maps are contoured at 2.5 σ and displayed 3.5 Å around the oligosaccharide ligands. In D, a simulated annealed omit electron density map, contoured at 0.7 σ, is shown in addition to the difference map and colored light green. Here, both maps are displayed 15 Å around the oligosaccharide ligand. The density in the lower part of the panel corresponds to the MCPyV VP1. Glycosidic linkages to other monosaccharides are shown as black arrows. The red arrow denotes absent electron density for a branch, indicating a non-branching Gal residue. The protein is shown in cartoon representation and colored grey, with one monomer highlighted in cyan. The carbohydrates are shown in stick representation. Nitrogen and oxygen atoms are colored blue and red, respectively, and carbon atoms are colored orange for 3SLN (A), light orange for DSL (B) and yellow for GD1a (C,D).
(TIF)

Figure S2 **Alignment of receptor-binding VP1 sequences from MCPyV isolates and MCPyV-like viruses from chimpanzees.** Complete VP1 sequences were retrieved from GenBank and aligned with Muscle. Identical residues are indicated with an asterisk. Residues contacting carbohydrate are shaded dark teal if identical and light teal if not identical.
(TIF)

Figure S3 **Additional ^1^H-NMR-Spectra of MCPyV VP1 and DSL.** (A) DSL reference. (B) DSL STD spectrum with MCPyV VP1. (C) TOCSY spectrum of internal Neu5Ac ring displaying H3-H6 resonances which also appear in the STD spectrum (vertical lines). (D) TOCSY spectrum of terminal Neu5Ac ring displaying H3-H6 resonances which do not appear in the STD spectrum. (E) TOCSY spectrum of internal Neu5Ac ring displaying H7-H9 resonances which also appear in the STD spectrum (vertical lines). (F) On-resonance frequency control (DSL STD spectrum without MCPyV VP1). Asterisks in (F) denote impurities.
(TIF)

Figure S4 **Saturation transfer NMR spectra of MCPyV VP1 and GT1b.** (A) GT1b reference. (B) GT1b STD spectrum with MCPyV VP1. Asterisks denote impurities in both spectra. The spectra were recorded with GT1b compound (1), but similar conclusions were obtained from spectra with compound (2).
(TIF)
