# Supplementary figures and images for: Structures of Merkel Cell Polyomavirus VP1 Complexes Define a Sialic Acid Binding Site Required for Infection
Source: PLoS Pathog. 2012 Jul 26;8(7):e1002738. doi: 10.1371/journal.ppat.1002738 (PMC3406085; doi:10.1371/journal.ppat.1002738)

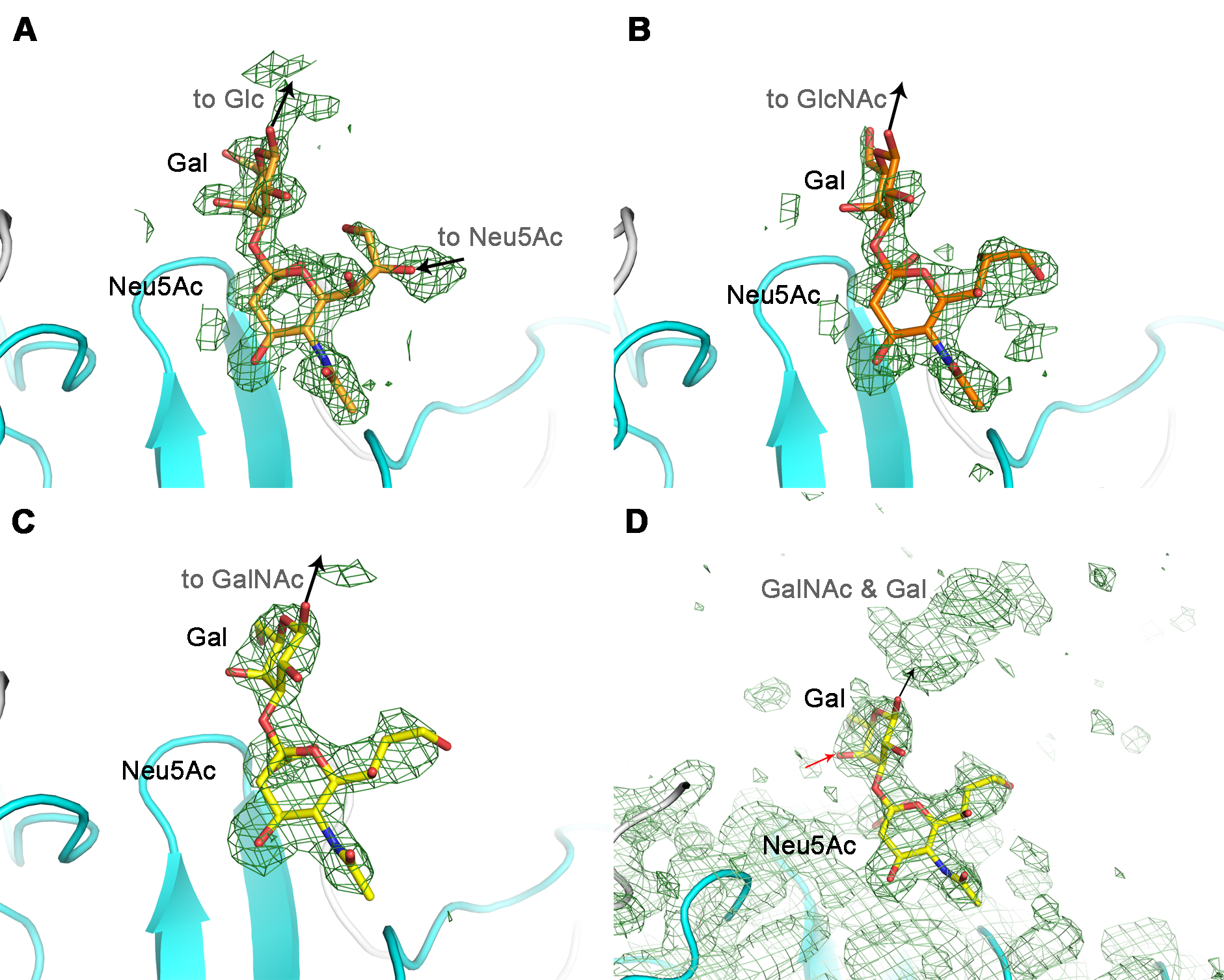

Supplement: Figure S1 — Electron density for carbohydrate ligands. Simulated annealed omit difference electron density maps for DSL (A), 3SLN (B) and GD1a (C,D). In A–C, the maps are contoured at 2.5 σ and displayed 3.5 Å around the oligosaccharide ligands. In D, a simulated annealed omit electron density map, contoured at 0.7 σ, is shown in addition to the difference map and colored light green. Here, both maps are displayed 15 Å around the oligosaccharide ligand. The density in the lower part of the panel corresponds to the MCPyV VP1. Glycosidic linkages to other monosaccharides are shown as black arrows. The red arrow denotes absent electron density for a branch, indicating a non-branching Gal residue. The protein is shown in cartoon representation and colored grey, with one monomer highlighted in cyan. The carbohydrates are shown in stick representation. Nitrogen and oxygen atoms are colored blue and red, respectively, and carbon atoms are colored orange for 3SLN (A), light orange for DSL (B) and yellow for GD1a (C,D). (TIF) [file ppat.1002738.s001.tif]

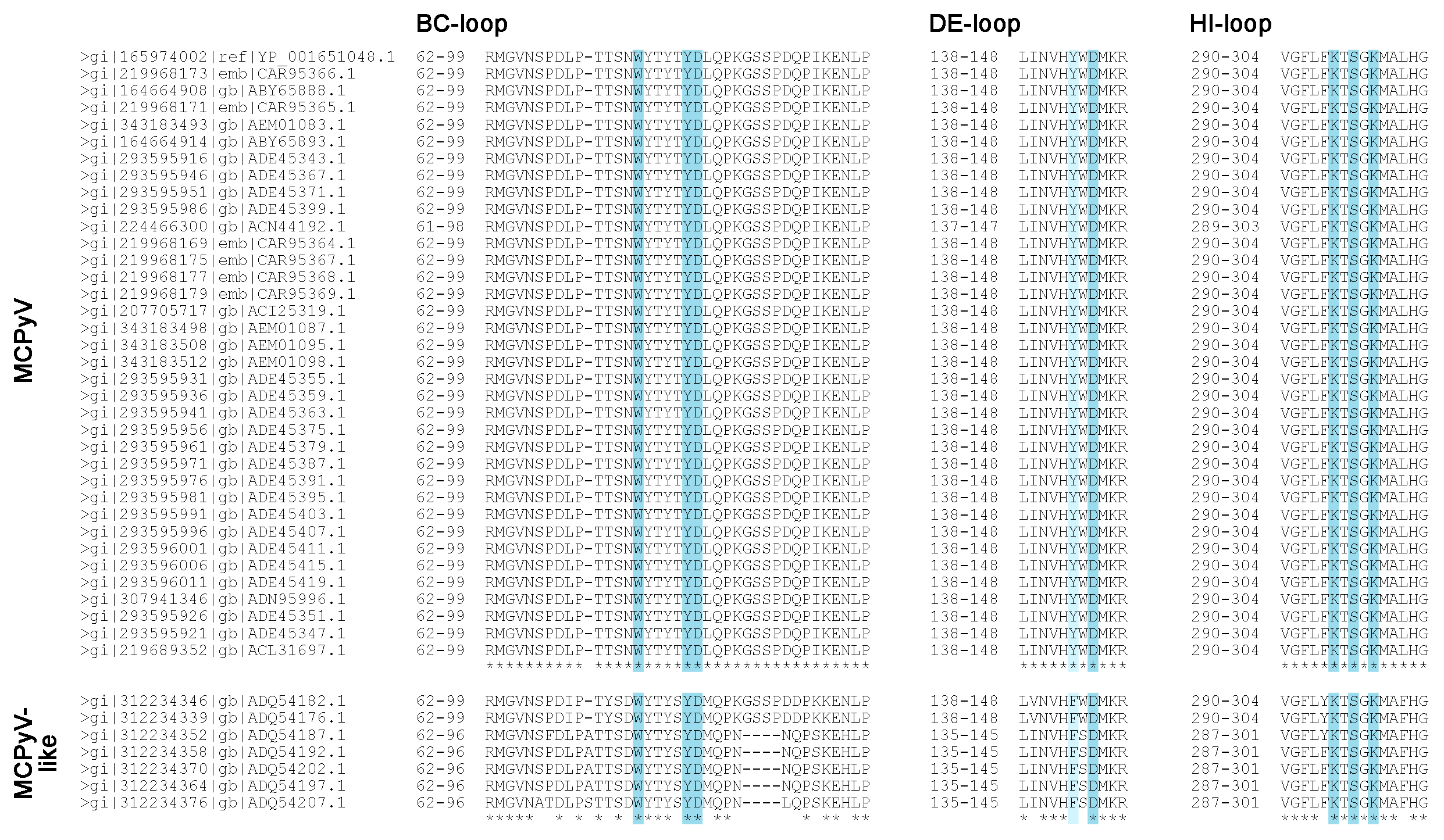

Supplement: Figure S2 — Alignment of receptor-binding VP1 sequences from MCPyV isolates and MCPyV-like viruses from chimpanzees. Complete VP1 sequences were retrieved from GenBank and aligned with Muscle. Identical residues are indicated with an asterisk. Residues contacting carbohydrate are shaded dark teal if identical and light teal if not identical. (TIF) [file ppat.1002738.s002.tif]

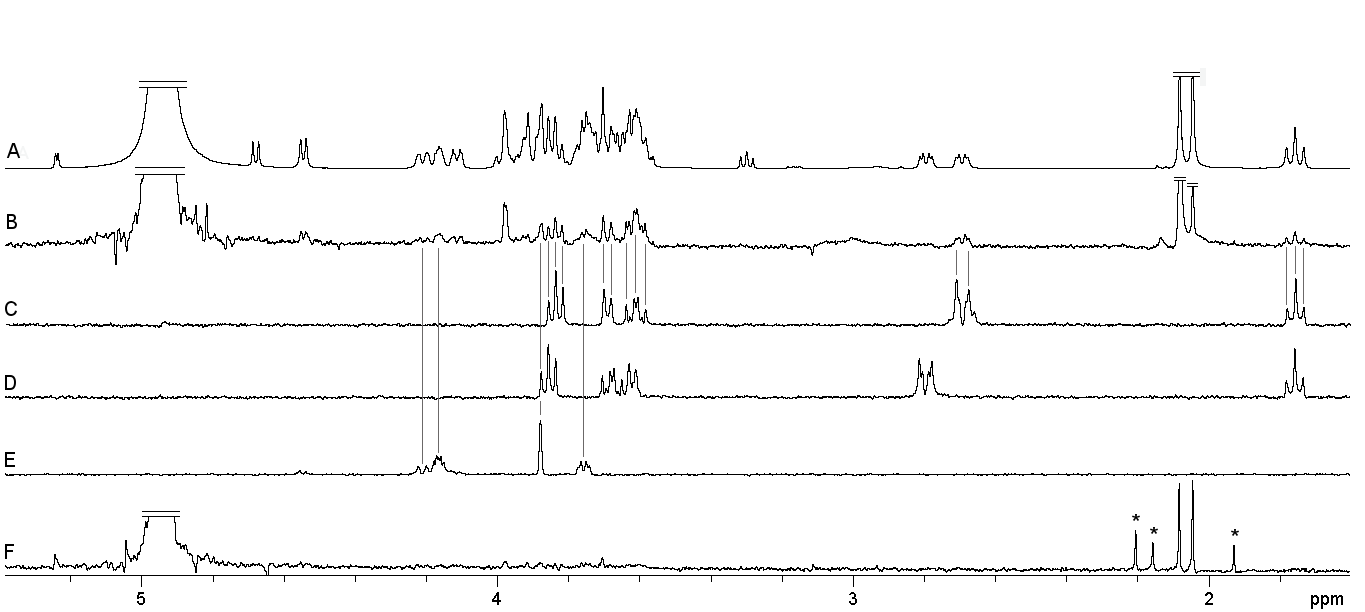

Supplement: Figure S3 — Additional 1H-NMR-Spectra of MCPyV VP1 and DSL. (A) DSL reference. (B) DSL STD spectrum with MCPyV VP1. (C) TOCSY spectrum of internal Neu5Ac ring displaying H3-H6 resonances which also appear in the STD spectrum (vertical lines). (D) TOCSY spectrum of terminal Neu5Ac ring displaying H3-H6 resonances which do not appear in the STD spectrum. (E) TOCSY spectrum of internal Neu5Ac ring displaying H7-H9 resonances which also appear in the STD spectrum (vertical lines). (F) On-resonance frequency control (DSL STD spectrum without MCPyV VP1). Asterisks in (F) denote impurities. (TIF) [file ppat.1002738.s003.tif]

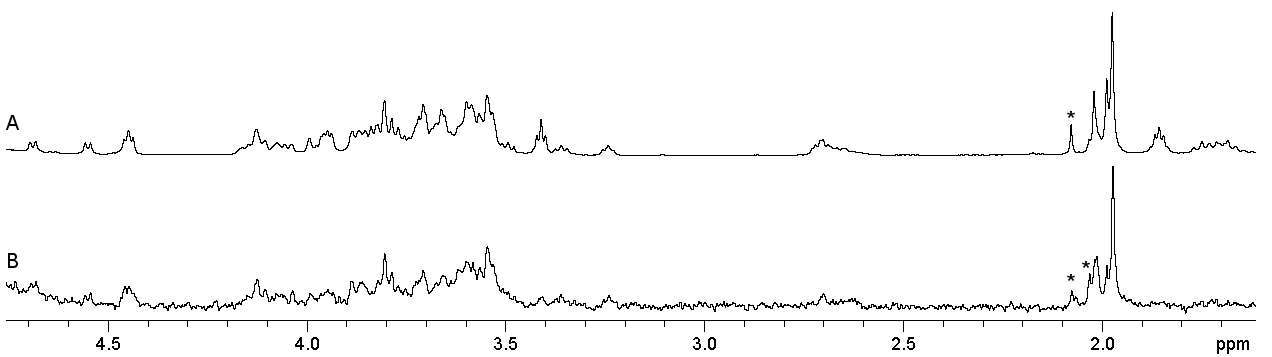

Supplement: Figure S4 — Saturation transfer NMR spectra of MCPyV VP1 and GT1b. (A) GT1b reference. (B) GT1b STD spectrum with MCPyV VP1. Asterisks denote impurities in both spectra. The spectra were recorded with GT1b compound (1), but similar conclusions were obtained from spectra with compound (2). (TIF) [file ppat.1002738.s004.tif]
